# Efficacy and Safety of Therapies for Pediatric Steroid-Resistant Idiopathic Nephrotic Syndrome: A Systematic Review of the Last Decade

**DOI:** 10.7759/cureus.101030

**Published:** 2026-01-07

**Authors:** Beatriz de Sousa, Joana Torres Ribeiro, Catarina Azevedo, Patrícia Sousa, Cláudia Tavares

**Affiliations:** 1 Department of Pediatrics, Unidade Local de Saúde do Alto Ave, Guimarães, PRT; 2 Department of Pediatrics, Escola de Medicina - Universidade do Minho, Braga, PRT

**Keywords:** childhood nephrotic syndrome, nephrotic syndrome treatment, pediatric nephrology, steroid resistance, steroid-resistant nephrotic syndrome

## Abstract

Steroid-resistant nephrotic syndrome (SRNS) represents a major therapeutic challenge in pediatric nephrology, being associated with poor prognosis and increased risk of progression to chronic kidney disease. Over the past decade, several therapeutic strategies have been evaluated, but evidence regarding their efficacy and safety remains heterogeneous. The main objective of this study is to systematically review the efficacy and safety of therapies for pediatric idiopathic SRNS.

We systematically searched PubMed/MEDLINE, SCOPUS, Web of Science, ScienceDirect, BMC Pediatrics, and Cochrane Library/CENTRAL for studies published between January 2014 and February 2024. Randomized controlled trials (RCTs), nonrandomized experimental studies, and prospective cohorts evaluating therapeutic interventions in children with idiopathic SRNS were included. Study selection, data extraction, and quality assessment were performed independently by two reviewers, using Joanna Briggs Institute (JBI) tools. The review protocol was registered in PROSPERO (CRD42024558619).

Out of 12,678 records identified, 10 studies fulfilled the eligibility criteria, comprising 441 pediatric patients. Five were RCTs, four non-randomized experimental studies, and one prospective cohort. Interventions evaluated included tacrolimus (TAC), cyclosporine A (CsA), mycophenolate mofetil (MMF), cyclophosphamide (oral and intravenous), leflunomide (LEF), rituximab (RTX), and ofatumumab (OFA), mostly in combination with steroids. TAC demonstrated higher remission rates compared with CsA and MMF, with fewer adverse effects. Triple immunosuppressive therapy (TAC + steroids + antimetabolite) improved both short- and long-term remission rates compared with two-drug (dual) regimens. RTX showed partial efficacy, reducing proteinuria and steroid burden, while OFA did not achieve significant benefit over placebo. MMF following RTX was associated with higher relapse-free survival compared with CsA. Oral and intravenous cyclophosphamide had similar efficacy and safety profiles. Across studies, adverse effects were predominantly steroid- or calcineurin inhibitor (CNI)-related, while severe events included infections, hematological toxicity, and rare drug-related nephrotoxicity.

Current evidence suggests TAC-based regimens offer superior efficacy and safety compared with CsA or MMF in pediatric idiopathic SRNS. Anti-CD20 monoclonal antibodies present variable results, with RTX showing limited but measurable benefit and OFA lacking efficacy. MMF appears favorable after RTX compared to CsA, while cyclophosphamide shows no advantage between oral and intravenous administration. Despite progress, evidence remains limited by small sample sizes and heterogeneity, underscoring the need for large-scale, multicenter trials to optimize therapeutic strategies.

## Introduction and background

Steroid-resistant nephrotic syndrome (SRNS) remains one of the most challenging glomerular disorders in pediatric nephrology. Idiopathic nephrotic syndrome (NS) is among the most common kidney diseases in childhood, clinically defined by nephrotic range proteinuria, hyperlipidemia, hypoalbuminemia, and edema [1-4]. The prevalence is ~16 per 100,000 children, with an incidence of 2-7 per 100,000 children/year, most frequently presenting between one and six years of age [1,3,5,6]. Proposed pathophysiology involves immune dysregulation with T-cell-mediated podocyte injury and circulating permeability factors, while monogenic forms (most commonly involving NPHS1, NPHS2, WT1, and LAMB2) account for a subset of cases, particularly at younger ages [3,7-9]. Early genetic testing is recommended for all children with SRNS who fail to respond or partially respond to immunosuppressants, as identification of a pathogenic mutation strongly predicts resistance to immunosuppressive therapy and may alter management. Morbidity is substantial, including infection (from urinary losses of complement and immunoglobulins) and thromboembolic complications due to a hypercoagulable state, as well as long-term cardiovascular sequelae and psychosocial burden for patients and families [3,5,10-12].

Primary idiopathic NS is most often caused by minimal change disease (MCD) and focal segmental glomerulosclerosis (FSGS), with the incidence of FSGS reportedly increasing over time [5,6,13]. Secondary causes include systemic disorders (e.g., lupus, vasculitis), infections (e.g., hepatitis B and C), malignancy, and drug-induced disease [3,5,6].

Oral corticosteroids remain the initial standard of care in idiopathic NS [3,5,9,14]. However, prolonged exposure is associated with growth impairment, obesity, osteopenia, hypertension, and cataracts [2,15-17].

Prognosis is largely determined by steroid response and relapse pattern in the first year; after ≥4-6 weeks of adequate therapy, NS is classified as steroid-sensitive or steroid-resistant [2,5,18,19]. Approximately 90% of first-episode NS cases remit with steroid therapy (about 95% by four weeks and ~98% by eight weeks). Cases with no remission or response by four to six weeks (steroid non-responders) should undergo a kidney biopsy; nearly half of these children show FSGS on histology, which is a leading cause of end-stage kidney disease (ESKD) in pediatric SRNS and may recur after transplantation [2,14,18-20].

Children who fail to respond to corticosteroids are treated with immunosuppressants, including calcineurin inhibitors (CNIs) (cyclosporine A (CsA) and tacrolimus (TAC)), cyclophosphamide (CYC), mycophenolate mofetil (MMF), and anti-CD20 monoclonal antibodies (e.g., rituximab) [2,4,9,21-30]. Yet, due to the rarity of SRNS, high-quality randomized controlled trials (RCTs) are difficult to conduct, and treatment algorithms lack robust evidence; practice patterns vary across centers, influenced by clinician preference, regional drug availability, and interpretation of limited data [3,5]. Given the clinical impact on growth, cardiovascular risk, infection, and quality of life for children and caregivers, defining the most effective and safest regimens is a priority [6,10-12,31,32]. Accordingly, the objective of this systematic review is to synthesize the last decade of evidence on the efficacy and safety of therapies for pediatric idiopathic SRNS to inform clinical decision-making and future research. Supportive measures such as renin-angiotensin system blockade, diuretics, and careful blood pressure control are considered standard of care in all patients with SRNS [2], but these will not be the focus of the present review.

## Review

Methodology

Study Type

This work was conducted as a systematic review following the Preferred Reporting Items for Systematic Reviews and Meta-Analyses (PRISMA) 2020 guidelines [33]. The review protocol was designed a priori, defining eligibility criteria, information sources, and outcomes of interest. The main goal was to evaluate the efficacy and safety of therapies for steroid-resistant idiopathic NS in the pediatric population. It aims to answer the research question: “What therapeutic advances in the last decade have been achieved in the treatment of children with idiopathic steroid-resistant nephrotic syndrome?” This systematic review is registered in the PROSPERO database under the registration number CRD42024558619. No separate publicly available protocol was prepared beyond the PROSPERO record. No amendments were made to the original protocol after registration.

Eligibility Criteria

We included RCTs, non-randomized experimental studies, and prospective cohort studies evaluating pharmacological therapies in pediatric patients (<18 years) with idiopathic SRNS. Studies were eligible if they reported outcomes related to treatment efficacy (complete or partial remission of proteinuria) and/or safety (adverse drug reactions). Exclusion criteria comprised: studies including adult populations; non-idiopathic or genetic SRNS without separate analysis; retrospective case series or reports; reviews, editorials, and conference abstracts; and articles not written in English or Portuguese.

Search Strategy

A comprehensive search [34] was performed in PubMed/MEDLINE, SCOPUS, Web of Science, ScienceDirect, BMC Pediatrics, and Cochrane Library/CENTRAL, covering the period from January 1, 2014, to February 1, 2024. Search terms included combinations of Medical Subject Headings (MeSH) and free text: (“steroid-resistant nephrotic syndrome” OR “SRNS”) AND (“children” OR “pediatric”) AND (“treatment” OR “therapy” OR “immunosuppressants” OR “rituximab” OR “tacrolimus” OR “cyclosporine” OR “mycophenolate” OR “cyclophosphamide”). Reference lists of included articles were screened manually to identify additional eligible studies. Each database’s specific search strategy and results are detailed in Table 1.

**Table 1:** Search strategy for the different databases

Database	Query	Filters	Search Strategy
Pubmed/MEDLINE	(Steroid-resistant nephrotic syndrome OR SRNS) AND (pediatric OR child OR children) AND (treatment OR therapeutics)	Publication date: 2014-2024; Article Type: Randomized controlled Trials; Clinical Trials	(("nephrotic syndrome"[MeSH Terms] OR ("nephrotic"[All Fields] AND "syndrome"[All Fields]) OR "nephrotic syndrome"[All Fields] OR ("steroid"[All Fields] AND "resistant"[All Fields] AND "nephrotic"[All Fields] AND "syndrome"[All Fields]) OR "steroid resistant nephrotic syndrome"[All Fields] OR ("nephrotic syndrome"[MeSH Terms] OR ("nephrotic"[All Fields] AND "syndrome"[All Fields]) OR "nephrotic syndrome"[All Fields] OR "srns"[All Fields])) AND ("paediatrics"[All Fields] OR "pediatrics"[MeSH Terms] OR "pediatrics"[All Fields] OR "paediatric"[All Fields] OR "pediatric"[All Fields] OR ("child"[MeSH Terms] OR "child"[All Fields] OR "children"[All Fields] OR "child s"[All Fields] OR "children s"[All Fields] OR "childrens"[All Fields] OR "childs"[All Fields]) OR ("child"[MeSH Terms] OR "child"[All Fields] OR "children"[All Fields] OR "child s"[All Fields] OR "children s"[All Fields] OR "childrens"[All Fields] OR "childs"[All Fields])) AND ("therapeutics"[MeSH Terms] OR "therapeutics"[All Fields] OR "treatments"[All Fields] OR "therapy"[MeSH Subheading] OR "therapy"[All Fields] OR "treatment"[All Fields] OR "treatment s"[All Fields] OR ("therapeutical"[All Fields] OR "therapeutically"[All Fields] OR "therapeuticals"[All Fields] OR "therapeutics"[MeSH Terms] OR "therapeutics"[All Fields] OR "therapeutic"[All Fields]))) AND ((clinicaltrial[Filter] OR randomizedcontrolledtrial[Filter]) AND (2014:2024[pdat]))
SCOPUS		Publication Date: 2014-2024; Document Type: Article	TITLE-ABS-KEY ( ((steroid AND resistant AND nephrotic AND syndrome) OR srns) AND (pediatric OR child OR children) AND (treatment OR therapeutics) ) AND PUBYEAR > 2013 AND PUBYEAR < 2025 AND (LIMIT-TO (DOCTYPE, "ar"))
Web of Science		Publication Date: 2014-2024; Document Type: Article	Results for ALL=((Steroid resistant nephrotic syndrome OR SRNS) AND (pediatric OR child OR children) AND (treatment OR therapeutics)) and Article (Document Types) and 2024 or 2023 or 2022 or 2021 or 2020 or 2019 or 2018 or 2017 or 2016 or 2015 or 2014 (Publication Years)
ScienceDirect		Publication date: 2014-2024; Document type: Research article	-
Bmc pediatrics		Search by keywords: Articles	(Steroid resistant nephrotic syndrome OR SRNS) AND (pediatric OR child OR children) AND (treatment OR therapeutics) - All volumes
Cochrane/CENTRAL		Title, Abstract, Keywords, 2014-2024, Trials	(Steroid resistant nephrotic syndrome OR SRNS) AND (pediatric OR child OR children) AND (treatment OR therapeutics) - Title Abstract Keyword - (Word variations have been searched)

Study Selection

Two independent reviewers screened titles and abstracts for eligibility. Full texts of potentially relevant studies were retrieved and assessed in duplicate. Disagreements were resolved by consensus or by consulting a third reviewer. The selection process is summarized in a PRISMA flowchart. Data were independently extracted by two reviewers using a standardized form, including study design, country, sample size, demographics, histological findings, interventions, co-interventions, follow-up duration, definitions of remission, efficacy outcomes, and adverse events. Extracted information was cross-checked, and discrepancies were resolved by discussion.

Risk of Bias and Quality Assessment

Risk of bias was assessed using the Joanna Briggs Institute (JBI) critical appraisal tools, according to study design [35]. RCTs were evaluated for randomization, allocation concealment, blinding, completeness of follow-up, reliability of outcome measurement, and statistical appropriateness [36]. Non-randomized experimental studies and prospective cohorts were assessed for comparability of groups, exposure/outcome measurement, confounding control, and completeness of follow-up [37]. Studies were classified as high, moderate, or low quality. No study was excluded based on risk of bias assessment. Publication bias was not evaluated due to the low number of RCTs available.

Data Synthesis

Due to heterogeneity in study designs, populations, interventions, and outcome definitions, quantitative meta-analysis was not feasible. Results are therefore presented as a structured qualitative synthesis, grouped by therapeutic class (CNIs, anti-CD20 monoclonal antibodies, and CYC).

Results

Study Selection

**Figure 1:**
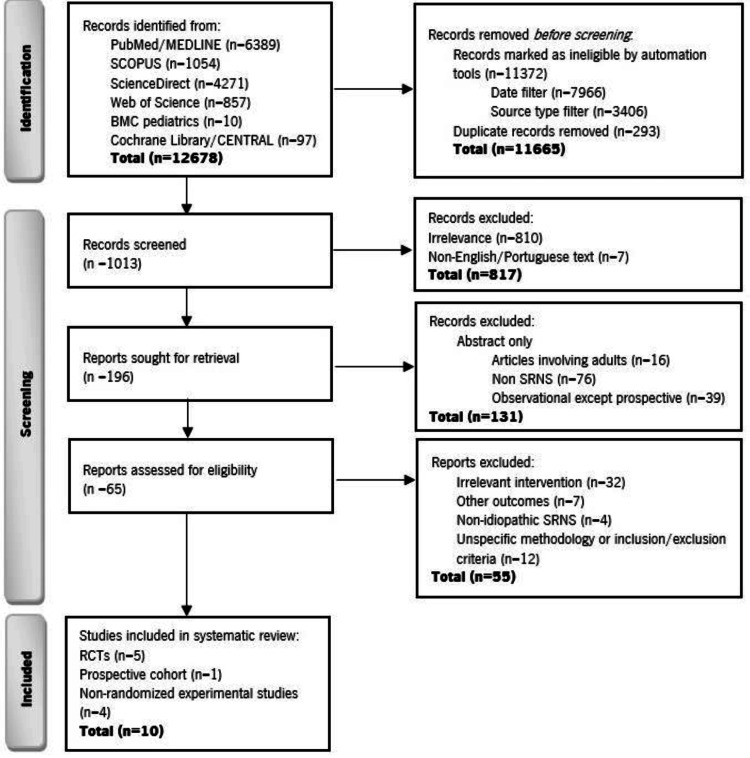
PRISMA flowchart Abbreviations: PRISMA, Preferred Reporting Items for Systematic Reviews and Meta-Analyses; SRNS, steroid-resistant nephrotic syndrome; RCT, randomized controlled trial

The database search initially identified 12,678 records (PubMed/MEDLINE, n = 6,389; SCOPUS, n = 1,054; ScienceDirect, n = 4,271; Web of Science, n = 857; BMC Pediatrics, n = 10; Cochrane Library/CENTRAL, n = 97). After automated filtering by date (January 2014-February 2024) and source type, and removal of duplicates (n = 293), 1,013 records were screened. Following title and abstract evaluation, 817 were excluded for irrelevance (n = 810) or language other than English/Portuguese (n = 7). A further 131 reports were excluded at the eligibility stage (adults included, n = 16; non-SRNS, n = 76; retrospective/non-prospective designs, n = 39). Of the 65 full-text articles assessed, 55 were excluded for irrelevant interventions (n = 32), outcomes (n = 7), non-idiopathic SRNS (n = 4), or insufficient methodological details (n = 12). Finally, 10 studies met the inclusion criteria: five RCTs, four non-randomized experimental studies, and one prospective cohort study [31,38-46]. The study selection process is illustrated in the PRISMA 2020 flow diagram (Figure 1).

Study Quality Assessment and Risk of Bias

All 10 studies underwent quality appraisal using the JBI tools [35]. Five RCTs achieved high scores, particularly regarding randomization, allocation concealment, baseline comparability, blinded outcome assessment, complete follow-up, and appropriate statistical analysis. The lowest scores were observed for participant and provider blinding. Among the four non-randomized experimental studies, two were rated high quality and two moderate, with limitations related to the absence of control groups and the lack of repeated outcome measurements. The single prospective cohort study scored high quality. None of the studies were excluded due to risk of bias concerns. Detailed quality assessment is provided in Tables 2-4.

**Table 2:** Risk of bias assessed using the Joanna Briggs Institute Critical Appraisal Checklist for randomized controlled trials Y - yes; N - no; Q - question Questions: 1) Was true randomization used for the assignment of participants to treatment groups?; 2) Was allocation to treatment groups concealed?; 3) Were treatment groups similar at the baseline?; 4) Were participants blind to treatment assignment?; 5) Were those delivering treatment blind to treatment assignment?; 6) Were outcomes assessors blind to treatment assignment?; 7) Were treatment groups treated identically other than the intervention of interest?; 8) Was follow-up complete, and if not, were differences between groups in terms of their follow-up adequately described and analyzed?; 9) Were participants analyzed in the groups to which they were randomized?; 10) Were outcomes measured in the same way for treatment groups?; 11) Were outcomes measured in a reliable way?; 12) Was an appropriate statistical analysis used?; 13) Was the trial design appropriate, and were any deviations from the standard RCT design (individual randomization, parallel groups) accounted for in the conduct and analysis of the trial? Quality Score: Low, 0-12; Moderate, 13-19; High, 20-26

	Q1	Q2	Q3	Q4	Q5	Q6	Q7	Q8	Q9	Q10	Q11	Q12	Q13	Quality Score
Wu et al. (2015) [38]	Y	Y	Y	N	N	Y	Y	Y	Y	Y	Y	Y	Y	High
Sinha et al. (2017) [41]	Y	Y	N	N	N	Y	Y	Y	Y	Y	Y	Y	Y	High
Shah and Hafeez (2016) [40]	Y	Y	N	N	N	Y	Y	Y	Y	Y	Y	Y	Y	High
Ravani et al. (2020) [43]	Y	Y	Y	Y	Y	Y	Y	Y	Y	Y	Y	Y	Y	High
Assadi et al. (2022) [45]	Y	Y	Y	N	N	Y	Y	Y	Y	Y	Y	Y	Y	High

**Table 3:** Risk of bias assessed using the Joanna Briggs Institute Critical Appraisal Checklist for quasi-experimental studies (non-randomized experimental studies) Y - yes; N - no; Q - question Questions: 1) Is it clear in the study what is the "cause" and what is the "effect" (i.e., there is no confusion about which variable comes first)?; 2) Were the participants included in any similar comparisons?; 3) Were the participants included in any comparisons receiving similar treatment/care, other than the exposure or intervention of interest?; 4) Was there a control group?; 5) Were there multiple measurements of the outcome, both pre- and post-intervention/exposure?; 6) Was follow-up complete, and if not, were differences between groups in terms of their follow-up adequately described and analyzed?; 7) Were the outcomes of participants included in any comparisons measured in the same way?; 8) Were outcomes measured in a reliable way?; 9) Was an appropriate statistical analysis used? Quality Score: Low, 0-8; Moderate, 9-13; High, 14-18

	Q1	Q2	Q3	Q4	Q5	Q6	Q7	Q8	Q9	Quality Score
Shah and Hafeez (2016) [40]	Y	Y	Y	N	N	Y	Y	Y	Y	High
Ahn et al. (2018) [31]	Y	Y	N	N	N	Y	Y	Y	Y	Moderate
Chen et al. (2020) [39]	Y	Y	N	N	N	Y	Y	Y	Y	Moderate
Nozu et al. (2024) [44]	Y	Y	Y	N	N	Y	Y	Y	Y	High

**Table 4:** Risk of bias assessed using the Joanna Briggs Institute Critical Appraisal Checklist for cohort studies Y - yes; N - no; Q - question Questions: 1) Were the two groups similar and recruited from the same population?; 2) Were the exposures measured similarly to assign people to both the exposed and unexposed groups?; 3) Was the exposure measured in a valid and reliable way?; 4) Were confounding factors identified?; 5) Were strategies to deal with confounding factors stated?; 6) Were the groups/participants free of the outcome at the start of the study (or at the moment of exposure)?; 7) Were the outcomes measured in a valid and reliable way?; 8) Was the follow-up time reported and sufficient to be long enough for outcomes to occur?; 9) Was follow-up complete, and if not, were the reasons for loss to follow-up described and explored?; 10) Were strategies to address incomplete follow-up utilized?; 11) Was appropriate statistical analysis used? Quality score: Low, 0-10; Moderate, 11-16; High, 17-22

	Q1	Q2	Q3	Q4	Q5	Q6	Q7	Q8	Q9	Q10	Q11	Quality Score
Prasad et al. (2018) [42]	Y	Y	Y	N	N	Y	Y	Y	Y	Y	Y	High

Study Characteristics

The 10 included studies comprised five RCTs (50%), four non-randomized experimental studies (40%), and one prospective cohort study (10%). Together, these studies evaluated a total of 441 children with idiopathic SRNS. The majority of participants were male (64.7%), with an overall male-to-female ratio of approximately 2:1.

Geographically, most studies were conducted in Asia, with India accounting for three (30%) and China for two (20%). Single studies were reported from Korea, Iran, Italy, Japan, and Pakistan (10% each). Across studies, populations were homogeneous, including only children with SRNS, although subgroups with resistance to CNIs, CYC, or MMF were variably represented.

Histopathological findings were reported in most studies, with FSGS being the most frequent lesion (40.5%), followed by MCD (28.2%), and mesangioproliferative glomerulonephritis (MPGN, 27.3%). Other histological subtypes were uncommon, each accounting for less than 2% of the study population.

Interventions were heterogeneous and included TAC, CsA, MMF, CYC (oral and intravenous), leflunomide (LEF), rituximab (RTX), and ofatumumab (OFA), most frequently in combination with corticosteroids. Follow-up periods ranged from six months to three years, and all studies assessed remission of proteinuria as the primary efficacy outcome, although definitions of complete and partial remission were not uniform across trials. Safety outcomes included treatment-related adverse events, reported in all studies with variable granularity.

Given the heterogeneity in outcome reporting across studies, particularly regarding steroid resistance and remission criteria, a comparative overview of the definitions is provided in Table 5.

**Table 5:** Definitions of steroid resistance, remission, and other outcome measures across the included studies Definitions of steroid resistance, remission, and other outcomes are reported verbatim from the included studies. Heterogeneity in criteria reflects differences in study design and was not standardized. Abbreviations: ACE, angiotensin-converting enzyme; ARB, angiotensin receptor blocker; CNI, calcineurin inhibitor; KDIGO, Kidney Disease: Improving Global Outcomes; MMF, mycophenolate mofetil; uPCR, urine protein-to-creatinine ratio.

	Steroid Resistance	Remission	Other Definitions
Wu et al. (2015) [38]	Failure to achieve remission after prednisone (or equivalent) at 1.5-2 mg/kg/day for more than 4 weeks, after exclusion of confounding causes such as infection, hypercoagulability, or thrombosis.	Short-term response (short-term remission): disappearance of clinical symptoms and negative urine protein on three consecutive occasions or proteinuria <4 mg/h/m² within 6 months after initiation of triple therapy. Long-term remission: absence of relapse and proteinuria < 4 mg/h/m² during 12 months of follow-up after achieving short-term response.	Tacrolimus-resistant: no improvement in clinical signs after 6 months at therapeutic tacrolimus trough levels (5-12 ng/mL), with persistent proteinuria ≥ +++ or > 40 mg/h/m² and serum albumin < 30-35 g/L. Tacrolimus-sensitive but frequently relapsing nephrotic syndrome: ≥ 3 relapses within 1 year or ≥ 2 relapses within 6 months after complete remission with tacrolimus. Relapse: recurrence of nephrotic-range proteinuria during follow-up.
Chen et al. (2020) [39]	Failure to achieve remission after more than 6 weeks of full-dose prednisone therapy.	Overall remission (partial and complete) as defined by KDIGO criteria.	Relapse: recurrence of proteinuria during follow-up (definition not quantitatively specified). No remission: absence of partial or complete remission according to KDIGO criteria.
Shah and Hafeez (2016) [40]	Persistent proteinuria despite 4 weeks of oral prednisolone at 60 mg/m²/day.	Complete remission: urine protein nil on dipstick or proteinuria <4 mg/m²/hour. Partial remission: urine dipstick + to ++ or proteinuria >4 and <40 mg/m²/hour.	No response: urine dipstick +3 or proteinuria >40 mg/m²/hour despite 3 months of appropriate therapy.
Sinha et al. (2017) [41]	Nephrotic-range proteinuria despite 8 weeks of prednisolone therapy (2 mg/kg/day for 4 weeks followed by 1.5 mg/kg on alternate days for 4 weeks), defined as 3–4+ proteinuria on dipstick and spot uPCR ≥ 2.0 mg/mg.	Complete remission: spot uPCR < 0.2 mg/mg, confirmed on two occasions, with absence of edema and serum albumin > 2.5 g/dL. Partial remission: spot uPCR 0.2-2.0 mg/mg, confirmed on two occasions, with absence of edema and serum albumin > 2.5 g/dL.	Non-response: spot uPCR >2.0 mg/mg with edema or serum albumin <2.5 g/dL. Relapse: 3-4+ proteinuria on dipstick for 3 consecutive days after remission. Recurrence of steroid resistance: failure to achieve remission despite 4 weeks of daily prednisolone for a relapse. Frequent relapses: ≥3 relapses in 12 months or ≥2 relapses in the first 6 months if associated with serious adverse events.
Prasad et al. (2018) [42]	Failure to achieve remission after prednisone 60 mg/m²/day for 4 weeks.	Complete remission: urinary protein-to-creatinine ratio <200 mg/g or < 1+ protein on urine dipstick for 3 consecutive days. Partial remission: >50% reduction in proteinuria from baseline with absolute uPCR 200-2000 mg/g.	Cyclophosphamide resistance: failure to induce remission after 12 weeks of therapy. CNI resistance at 6 months: persistence of proteinuria >50% from baseline or uPCR > 2000 mg/g after 6 months of therapy. CNI resistance at 12 months: inability to induce complete remission. Relapse after CNI withdrawal: recurrence of uPCR ≥2000 mg/g or ≥3+ proteinuria on dipstick for 3 consecutive days.
Ahn et al. (2018) [31]	Failure to achieve remission despite continuous use of steroids and calcineurin inhibitors for >3 months.	Overall remission (partial and complete) as defined by KDIGO criteria.	Drug-dependent nephrotic syndrome: steroid and CNI dependence is defined as 2 consecutive relapses during therapy or within 2 weeks of discontinuation of the respective drug. Drug-resistant nephrotic syndrome: failure to achieve remission despite >3 months of continuous steroid and CNI therapy. Relapse: defined according to the KDIGO guideline.
Ravani et al. (2020) [43]	Failure to achieve remission after 6 weeks of prednisone at 60 mg/m²/day.	Complete remission: uPCR < 200 mg/g for 3 consecutive days. Partial remission: ≥ 50% reduction in proteinuria from baseline or absolute uPCR between 200 and 2000 mg/g for 3 consecutive days.	Multidrug-resistant nephrotic syndrome: resistance to steroids plus resistance to calcineurin inhibitors and MMF. CNI resistance: failure to achieve complete remission within 6 months of cyclosporine or tacrolimus therapy at target trough levels. MMF resistance: failure to achieve complete remission after ≥ 6 months of MMF therapy (1200 mg/m²/day).
Nozu et al. (2023) [44]	Failure to achieve remission within 4 to 8 weeks of glucocorticoid treatment.	Complete remission: negative early-morning urine protein on dipstick for 3 consecutive days or urinary protein-to-creatinine ratio (Up/Uc) < 0.2 g/g creatinine for 3 consecutive days. Incomplete (partial) remission: early-morning urine protein ≥ 1+ on dipstick or Up/Uc ≥ 0.2 g/g creatinine with serum albumin > 2.5 g/dL (avaliado no dia 169).	Multidrug-resistant nephrotic syndrome: resistance to calcineurin inhibitors and failure to respond to ≥ 3 courses of steroid pulse therapy, with persistent nephrotic status.
Assadi et al. (2022) [45]	Lack of complete remission within 4 to 6 weeks of prednisone at standard dose (60 mg/1.73 m²/day or 2 mg/kg/day, maximum 60 mg/day), with no further response to three intravenous methylprednisolone pulses (500 mg/m² or 15 mg/kg) in conjunction with ACE inhibitors or ARBs.	Complete remission: urine dipstick protein nil or trace for 3 consecutive days or urine protein/creatinine ratio < 0.2 mg/mg. Partial remission: urine protein/creatinine ratio > 0.2 and < 2.0 mg/mg.	Relapse: urine dipstick protein ≥ 2+ for 3 consecutive first-morning urine samples or urine protein/creatinine ratio ≥ 2.0 mg/mg. Treatment failure: ≥ 2 relapses within 6 months.
Shah et al. (2017) [46]	Failure to achieve remission after 4 weeks of daily prednisolone at 60 mg/m².	Complete remission: urine protein nil or trace on at least 3 consecutive days or uPCR < 0.5. Partial remission: urine protein excretion < 2+ or uPCR 0.5-2.0 with serum albumin > 2.5 g/dL.	No response: persistence of 3+ or 4+ proteinuria or uPCR > 2.0 (nephrotic-range proteinuria).

The key characteristics of the included studies, including design, setting, population, interventions, and outcomes evaluated, are summarized in Table 6.

**Table 6:** Key characteristics of the selected studies * Only the single-arm trial was included in this work. Abbreviations: CNI, calcineurin inhibitor; CR, complete remission; CsA, cyclosporine A; CYC, cyclophosphamide; DMH, diffuse mesangial hypercellularity; DRNS, drug-resistant nephrotic syndrome; FSGS, focal segmental glomerulosclerosis; IgMN, IgM nephropathy; IV, intravenous; IV CYC (IVCP), intravenous cyclophosphamide; LEF, leflunomide; MCD, minimal change disease; MMF, mycophenolate mofetil; MPGN, membranoproliferative glomerulonephritis; MRNS, multidrug-resistant nephrotic syndrome; MRSN, multidrug-resistant steroid-resistant nephrotic syndrome; NR, no response; OCP, oral cyclophosphamide; OFA, ofatumumab; PR, partial remission; RCT, randomized controlled trial; RTX, rituximab; SRNS, steroid-resistant nephrotic syndrome; TAC, tacrolimus; Up/Uc, urinary protein-to-creatinine ratio

Study	Country	Design	Sample	Population	Treatment Groups - Biopsy	Intervention	Other interventions	Outcome	Follow-up
Triple Immunosuppressive Therapy With Tacrolimus									
Wu et al. (2015) [38]	China	RCT	Total N = 18: CYC group, 6; MMF group, 5; LEF group, 7	SRNS: TAC-resistant (n = 10), TAC-sensitive but frequently relapsing (n = 8)	CYC group: MCD, 2; IgMN, 2; FSGS, 2; MPGN, 0. MMF group: MCD, 3; IgMN, 0; FSGS, 1; MPGN, 1. LEF group: MCD, 5; IgMN, 0; FSGS, 2; MPGN, 0	Triple Therapy - Pre-randomization therapy: CYC (8-12 mg/kg/day for 2 days, then repeated at 2-4-week intervals) for 3-6 months, or MMF (20-30 mg/kg/day) for 12 months, or LEF (0.5-0.6 mg/kg/day for 2 days, then 0.2 mg/kg/day) for 12 months	Pre-randomization (Dual therapy): TAC (50-150 μg/kg/day) + Prednisone (1.5-2 mg/kg/day)	Relapse-free period	6-12 months
Tacrolimus and TAC Compared With Cyclosporine or Mycophenolate Mofetil									
Chen et al. (2020) [39]	China	Prospective non-randomized, non-controlled clinical trial	Total N = 76	SRNS	MPGN: 52, FSGS: 17, IgMN: 4, MCD: 1, C1qN: 1, MN:1	TAC (0.1 mg/kg/day)	Prednisone (0.5 mg/kg/day) until remission. Then tapered to 0.25 mg/kg/day. If persistently low blood TAC trough concentrations (<5 ng/mL): Diltiazem (3-6 mg/kg/day, <360 mg/day)	Overall remission rate (CR + PR) and NR at 1, 3, and 6 months	1-36 months
Shah and Hafeez (2016) [40]	Pakistan	Quasi-experimental	Total N = 84; TAC group: 42 and CsA group: 42	SRNS	TAC group: MCD, 2; FSGS, 9; MPGN, 31. CsA group: MCD, 6; FSGS, 9; MPGN, 27	TAC (0.1-0.2 mg/kg/day), or CsA (150-200 mg/m²/day)	Oral steroids (30 mg/m²/day) for 1 month, then every other day on a tapering dosage	CR, PR, or NR at 3 and 6 months	6 months
Sinha et al. (2017) [41]	India	Open-label RCT	Total N = 60; TAC group: 31 and MMF group: 29	SRNS	TAC group: MCD: 17 and FSGS: 14. MMF group: MCD: 17 and FSGS: 12	TAC (0.15 mg/kg/day) or MMF (750-1000 mg/m^2^/day)	Pre-randomization: Prednisolone (1.5 mg/kg/day), TAC (0.1-0.15 mg/kg/day), Enalapril (0.3-0.4 mg/kg/day), and Calcium Carbonate (250-500 mg/day), then Prednisolone (0.2-0.3 mg/kg, alternate days)	CR, PR, or NR at 12 months	12 months
Prasad et al. (2018) [42]	India	Prospective cohort	Total N = 45: TAC group: 22 and CSA group: 23	CYC-SRNS	CSA group: MCD: 7, FSGS: 16. TAC group: MCD: 7, FSGS: 15	0-6 months: CSA (5 mg/kg/day) OR TAC (0.1 mg/kg/day). 6-12 months: Response - continues on the same CNI. No response to the 1st CNI - switch to the other CNI group. After 12 months: - response - stop CNI. No response - MMF (1200 mg/m^2^) for 12 months	Prednisolone (0.5 mg/kg/day), Enalapril (0.2 mg/kg/day, max 20 mg/day if needed to control hypertension), Calcium Carbonate and Vitamin D, Nifedipine (10-30 mg) thrice a day if still hypertensive	Remission (either PR or CR) at 6 and 12 months of the 1st CNI	24 months
Anti-CD20 Monoclonal Antibodies									
Ahn et al. (2018) [31]	Korea	RCT and single-arm study*	Total N = 23	Single arm: DRNS (steroid- and CNI-resistant)	MCD: 8, FSGS: 10, C1qN: 2, Others: 2, Not performed: 1	Single dose of IV RTX (375 mg/m^2^, max. of 500 mg)	Pre-enrollment treatment with Steroids + CNI. Once remission was achieved, the steroid dose was reduced by 25% every 4 weeks, followed by CNI tapering by 25% every 4 weeks	Remission rate at 6 months	12 months
Ravani et al. (2020) [43]	Italy	Double-blind RCT	Total N = 13 - OFA group: 7. Control group: 6	MRSN (Steroid, CNI, and MMF Resistance)	Control group - MCD: 2, FSGS: 2, IgMN: 2. OFA group - MCD: 2, FSGS: 5, IgMN: 0	OFA arm: Single pulse of OFA (1500 mg/1.73 m^2^). Placebo arm: 1 L normal saline	Pretreatment: IV methylprednisolone (2 mg/kg) + oral paracetamol (15 mg/kg) + IV cetirizine (0.4 mg/kg)	CR or PR at 3 months	12 months
Nozu et al. 2024 [44]	Japan	Single-arm clinical trial	Total N = 6	MRNS (CNI + steroid)	MCD: 1, FSGS: 4, MPGN: 1	RTX (four 375-500 mg/m^2^ doses) at weekly intervals	Baseline CsA + steroid pulses administered 3 consecutive days (IV methylprednisolone succinate sodium 30-1000 mg/kg/day) + Baseline Prednisone (15-30 mg/m^2^/day). Premedication: acetaminophen, D-chlorpheniramine maleate, and methylprednisolone administered 30 minutes before the RTX infusion	>50% reduction in the Up/Uc from baseline on day 169	30 months
MMF vs. CsA (Post-rituximab)									
Assadi et al. (2022) [45]	Iran	Single-blinded RCT	Total N = 66 -RTX/CsA group: 34. RTX/MMF group: 32	SRNS	CsA group: MCD: 8, FSGS: 24, DMH: 2. MMF group: MCD: 7, FSGS: 23, DMH: 2	RTX infusions (375 mg/m^2^) and then CsA (2.5 mg/kg twice daily), or MMF (0.5 g/m^2^ twice daily)	Doses of prednisolone were reduced by 25 mg/kg/week and discontinued at the end of 6 weeks if patients were using prednisolone prior to study entry. All patients were off steroids at the time of recruitment	Relapse-free survival (calculated from patients who achieved CR) at 12 months	12 months
Intravenous vs. Oral Cyclophosphamide									
Shah et al. (2017) [46]	India	RCT	Total N = 50 - IV CYC group: 25, Oral CYC group: 25	SRNS	IVCP group: MCD: 15, FSGS: 5, MPGN: 5. OCP group: MCD: 14, FSGS: 8, MPGN: 3	Oral CYC (2 mg/kg/day) for 12 weeks or IV CYC (500 mg/m²/month) for 6 months	Alternate-day steroids in tapering doses. IV CYC group: Pretreatment: hydration + Ondansetron	CR, PR, or NR at the end of treatment	6 months

Comparison of immunosuppressive strategies

Triple Immunosuppressive Therapy With TAC

One RCT evaluated the addition of an antimetabolite (MMF, CYC, or LEF) to standard dual therapy (TAC + steroids). While dual therapy achieved complete remission in eight patients, triple therapy significantly improved both short-term remission (14/18, p = 0.043) and long-term relapse-free remission (11/14, p = 0.001). No significant differences were observed between antimetabolites. Adverse events were mainly steroid-related, with three cases of TAC-associated acute kidney injury, which resolved after dose adjustment [38].

TAC Compared With CsA or MMF

Four studies evaluated TAC in comparison to CsA or MMF. One non-randomized study combining TAC with steroids reported remission in more than 90% of patients within six months, with sustained non-nephrotic proteinuria and relapse rates between 28.9% and 39.7% over three years [31]. In direct comparisons, TAC achieved higher complete remission than CsA (97.6% vs. 80.9%, p = 0.014) [42]. Compared with MMF, TAC showed superior remission rates (90.3% vs. 44.8%, p = 0.0002), fewer relapses (p = 0.001), and reduced steroid dependence [41]. CsA was associated with hypertrichosis, gum hypertrophy, renal dysfunction, and septicemia-related deaths, while TAC generally caused fewer adverse effects. Severe infections and isolated deaths occurred in both groups [40,42].

Anti-CD20 Monoclonal Antibodies

Three studies evaluated monoclonal antibodies [31,43,44]. RTX, administered with CNIs and steroids, led to partial or complete remission in up to 43% of patients at nine months, with reduced proteinuria and lower steroid requirements, though without significant improvement in estimated glomerular filtration rate (eGFR) or hypertension [43,44,47]. OFA, by contrast, failed to achieve remission, with no meaningful change in proteinuria, serum albumin, or GFR compared with placebo [43]. RTX was associated with infusion reactions and infections, with one death attributed to refractory disease rather than treatment [24,48-53]. OFA was associated with mild infusion reactions and one death from pulmonary embolism, not considered drug-related [43].

MMF Compared to CsA (Post-RTX)

In one RCT, MMF was superior to CsA when administered after RTX, with higher relapse-free survival (81.3% vs. 61.7%, p = 0.02), shorter time to remission (2.6 vs. 3.4 months, p = 0.03), and greater efficacy in patients with FSGS (p = 0.02). MMF was also associated with fewer adverse effects (59.3% vs. 76.4%, p = 0.03), with hematological toxicity and infections more common in the CsA group [45].

Intravenous Versus Oral CYC

One RCT compared IV with oral CYC. Complete remission rates were similar (52% vs. 44%), though time to remission was significantly longer in the IV group (86 vs. 47 days, p = 0.002). Adverse events, including major infections, were observed in both groups, with no significant differences. Four patients progressed to renal failure, and one death occurred due to progressive kidney disease, not directly attributed to treatment [46].

Discussion

This systematic review synthesized evidence from the past decade regarding pharmacological interventions for idiopathic SRNS in children. Ten studies were identified, comprising five RCTs, four non-randomized experimental studies, and one prospective cohort, with a total of 441 patients. Overall, TAC-based regimens generally showed superior efficacy and tolerability compared with CsA or MMF [38,39,41,42,46,54], while the evidence for anti-CD20 monoclonal antibodies, particularly RTX, was heterogeneous [31,44]. OFA failed to demonstrate a significant benefit in the only available RCT [43]. MMF showed some utility, mainly as maintenance therapy after RTX-induced remission [45]. CYC offered no clear advantage between intravenous and oral administration [46].

Comparison With Previous Reviews and Guidelines

Our findings are consistent with earlier meta-analyses and systematic reviews. Li et al. concluded that TAC was more effective than CYC, MMF, or RTX in achieving remission, with fewer adverse effects [55]. Similarly, Liu et al. showed that CNIs were superior to CYC or placebo in inducing remission, although that review also included adult populations [29]. The present work updates the evidence by incorporating four more recent pediatric studies [39,43-45], strengthening the conclusion that TAC is the most effective first-line CNI. While TAC has shown a tendency towards higher remission rates and a more favorable safety profile compared with CsA, both CNIs are considered equally effective first-line therapies for idiopathic SRNS in current international guidelines, including the KDIGO guidelines [2] and the IPNA clinical practice recommendations [4].

TAC Versus Other Agents

Both RCTs and observational studies consistently support TAC as more effective than CsA in inducing remission [27,29,40,42,55]. Beyond efficacy, TAC appears to carry a lower risk of nephrotoxicity and fewer cosmetic adverse effects such as gingival hypertrophy and hypertrichosis [30,56-58]. When compared to MMF, TAC also demonstrated superior maintenance of remission, with significantly fewer relapses and lower corticosteroid requirements [41]. Triple immunosuppressive therapy combining TAC, steroids, and an antimetabolite achieved significantly better short- and long-term remission rates compared to dual therapy [38]. Although evidence is limited, these results suggest that selected patients may benefit from multi-drug regimens, provided safety is closely monitored.

Anti-CD20 Monoclonal Antibodies

RTX has emerged as a therapeutic option for children with CNI-resistant SRNS. In the studies included, RTX achieved partial or complete remission in up to 43% of patients, reduced proteinuria, and facilitated corticosteroid tapering [24,31,44,51,59,60]. However, responses were inconsistent, and the benefits on long-term renal function remain uncertain. These results contrast with the stronger evidence supporting RTX in steroid-dependent or frequently relapsing NS [24,52]. OFA, despite promising early reports [49,61], failed to demonstrate efficacy in the only RCT conducted in pediatric SRNS [43].

CYC and Other Agents

CYC has long been used in SRNS, but evidence supporting its role is weak. A retrospective five-year follow-up study comparing CYC and CsA in children with SRNS demonstrated superior long-term efficacy of CsA in maintaining remission [62]. In this review, IV and oral regimens showed similar efficacy and safety [46,63]. Given the risk of gonadal toxicity, malignancy, and other long-term adverse effects [22,23], current international guidelines discourage the use of CYC in idiopathic SRNS due to its low remission rates and significant toxicity; the IPNA 2020 [4] recommendations advise against oral CYC and only allow intravenous administration in exceptional cases when CNIs are unavailable, and clinical studies have consistently shown inferior efficacy compared to CNIs. Other agents such as MMF may still have value as maintenance therapy, particularly after RTX-induced remission [45,64].

Variability in Outcome Definitions

A key methodological issue is the lack of standardization in definitions of remission and relapse across trials. While most studies distinguished between complete and partial remission, thresholds varied, particularly for partial remission and relapse frequency. Some required a 50% proteinuria reduction, while others used strict urine protein-to-creatinine ratio cutoffs. This variability complicates cross-trial comparisons and reduces the feasibility of meta-analysis. Standardized outcome definitions are urgently needed to improve comparability and allow aggregation of results in future reviews.

Limitations of the evidence

This review has several limitations, largely inherent to the primary studies. Most were single-center and underpowered, with heterogeneous designs and populations [28,29,54]. Short follow-up times preclude conclusions about long-term renal survival or adverse effects. Genetic testing for monogenic SRNS was inconsistently performed [8], limiting insights into differential treatment response. Furthermore, several trials combined therapies, making it difficult to isolate drug-specific effects [9,21]. The available RCTs are limited by small sample sizes, heterogeneous populations (initial SRNS versus multidrug-resistant forms), and frequent open-label designs, all of which restrict the generalizability of their findings [31,38,40,45,53,55]. Additionally, a degree of publication bias cannot be excluded, as positive studies are more likely to be published, and some negative or inconclusive findings may not have entered the literature [29].

Limitations of the review process

Beyond the methodological limitations of the included studies, this review has some process-related constraints. Only studies published in English or Portuguese between 2014 and 2024 were considered, which may have excluded relevant reports in other languages or outside this timeframe. Grey literature and trial registries were not systematically searched, potentially missing unpublished data. Finally, the absence of a quantitative meta-analysis limits the ability to derive pooled estimates of treatment effect.

Future research

Future studies should prioritize adequately powered, multicenter RCTs with standardized outcome definitions and longer follow-up. Incorporating genetic and molecular stratification into trial design will be critical to advancing personalized therapy in SRNS. Trials evaluating combination regimens with agents of complementary mechanisms may provide further insights, as might studies exploring novel biologics targeting B cells, T cells, or cytokine pathways [65-70]. Beyond clinical remission, outcomes such as kidney survival, quality of life, and treatment burden - for both patients and families - warrant systematic evaluation.

## Conclusions

SRNS represents a small but high-risk subgroup of pediatric NS and is associated with poor response to standard therapy and an increased risk of progression to chronic kidney disease. Despite its relatively low prevalence, SRNS carries a substantial clinical and psychosocial burden, highlighting the need for effective, safe, and individualized therapeutic strategies.

Current evidence supports CNIs combined with corticosteroids as first-line therapy. CNIs (TAC or CsA) remain first-line; TAC may be favored in some settings, but comparative evidence remains limited and heterogeneous. MMF may be considered in CNI-resistant or CNI-dependent SRNS, while anti-CD20 agents, such as RTX and OFA, may be options in multidrug-resistant disease, although evidence remains limited. CYC is not recommended for routine use due to modest efficacy and long-term toxicity. Future studies should focus on well-designed comparative trials, standardized outcome definitions, and genetic stratification to improve treatment algorithms and patient-centered care in SRNS.
